# An Intelligent Music Production Technology Based on Generation Confrontation Mechanism

**DOI:** 10.1155/2022/5083146

**Published:** 2022-02-10

**Authors:** Yanjing Li, Xinyuan Liu

**Affiliations:** ^1^Department of Music, Langfang Normal University, Langfang, Hebei 065000, China; ^2^Department of Electronic and Information Engineering, Langfang Normal University, Langfang, Hebei 065000, China

## Abstract

In recent years, with the development of deep neural network becoming more and more mature, especially after the proposal of generative confrontation mechanism, academia has made many achievements in the research of image, video and text generation. Therefore, scholars began to use similar attempts in the research of music generation. Therefore, based on the existing theoretical technology and research work, this paper studies music production, and then proposes an intelligent music production technology based on generation confrontation mechanism to enrich the research in the field of computer music generation. This paper takes the music generation method based on generation countermeasure mechanism as the research topic, and mainly studies the following: after studying the existing music generation model based on generation countermeasure network, a time structure model for maintaining music coherence is proposed. In music generation, avoid manual input and ensure the interdependence between tracks. At the same time, this paper studies and implements the generation method of discrete music events based on multi track, including multi track correlation model and discrete processing. The lakh MIDI data set is studied. On this basis, the lakh MIDI is pre-processed to obtain the LMD piano roll data set, which is used in the music generation experiment of MCT-GAN. When studying the multi track music generation based on generation countermeasure network, this paper studies and analyzes three models, and puts forward the multi track music generation method based on CT-GAN, which mainly improves the existing music generation model based on GAN. Finally, the generation results of MCT-GAN are compared with those of Muse-GAN, so as to reflect the improvement effect of MCT-GAN. Select 20 auditees to listen to the generated music and real music and distinguish them. Finally, analyze them according to the evaluation results. After evaluation, it is concluded that the research effect of multi track music generation based on CT-GAN is improved.

## 1. Introduction

As an important way of expression in the field of art, music embodies a series of human unique thinking modes and is a unified combination of regularity and creativity [1]. On the one hand, the composition of music is naturally based on certain music theory rules, such as melody, rhythm, mode, chord, harmony, polyphony, musical form [2]. Music that cannot meet the constraints of music theory rules is often poor in auditory sweetness and cannot be accepted by the public [3]. On the other hand, music that simply meets the constraints of music theory is not necessarily good music. Music itself also acts as an important task of emotional expression carrier [4]. This requires the Creator not to stack the rules in a conventional way, but to incorporate innovation into the music, so that the generated melody will not be stereotyped and the mode is fixed. At the same time, the automatic generation of music by computer algorithm has always been a field of concern. Music creation with the help of computer algorithm can reduce the production threshold and save manpower and time cost [5]. To a certain extent, avoid the copyright problem, and quickly make a large number of music according to the scene needs, such as a large number of customized melodies that render the plot emotion in film and television dramas [6]. Due to the regularity and creativity of music and other artistic creation fields, the research on intelligent music creation can well measure and test the performance of artificial intelligence. When describing the experimental results of their own model algorithms, many related works in the field of music generation use the test method of organizing volunteers for auditory recognition [7]. The investigation and statistics are carried out from the aspects of authenticity, ear pleasing, creativity and interest. For example, the literature randomly looks for 255 testers to test the quality of music samples generated by their Muse-GAN model [8]. Similarly, some other algorithmic composition work also adopts this human judgment method. Generally speaking, the production process of intelligent music needs to minimize the workload of human intervention [9]. This production method can generate music automatically or semi automatically. The output results should not only meet the basic prior knowledge of music theory, but also have some algorithm creativity. The literature discusses that this process is to independently produce continuous audio signals or discrete symbol sequences from the computational model, and these signals and sequences must meet the music theory architecture [10]. As early as the 1950s, artificial intelligence technology was just in its infancy. Although limited by data and hardware performance, people also began to explore the field of intelligent composition and achieved some results [11]. In the early stage, intelligent music was mainly generated in two ways. First, it was created based on statistical analysis and combined with Markov chain models. For example, the literature was the first to create String Quartet suite with large-scale computer, which became the first music work completely generated by computer in history [12]. It used Markov chain model to generate random notes with limited control, combined with the rules of harmony and polyphony, these notes are tested, and the tested elements are modified to synthesize the string quartet of traditional music notation. The second is simple pattern matching and machine learning based on music theory rules, such as integrating music theory into machine learning to produce notes [13]. In recent years, deep learning has attracted more and more attention. Researchers have frequently applied deep learning technology in many fields and achieved good results. Therefore, some fields have produced projects that can be put into practical use. Based on this, people's research on neural network is emerging again. With the improvement of computer hardware technology, the deep learning technology based on neural network can not only deal with huge data samples, but also have strong computing power. Because of this, a series of problems such as many model parameters and difficult training have been solved, which are widely used to solve problems in various research fields. For example, deep learning technology greatly improves the accuracy of image classification, even exceeds human classification ability, and has successful applications in the field of natural language processing. At present, the generative countermeasure network based on neural network has been widely used in the fields of image, vision, voice and language. Therefore, this paper proposes to realize the automatic annotation of music based on generative countermeasure network.

With the continuous evolution and updating of AI technology, the formal technology of music creation has been further developed [14]. In recent years, generative countermeasure network has attracted more and more attention in the field of data generation. Relying on its strong fitting ability and simple training derivation process, it has been introduced into many application fields [15]. Generative countermeasure network has been widely used in graphics generation, graphics compression, speech generation and super-resolution restoration. The literature points out that “GAN can also generate text, dialogue generation, machine translation, voice generation, etc. At the same time, GAN is also involved in other fields, such as music generation, password decoding [16]. However, the application effect of GAN in other fields is not significant, so how to improve the application effect of GAN in other fields will be worthy of in-depth research, so as to make the generated countermeasure network shine in artificial intelligence “ [17]. The research on speech synthesis and music creation using GAN has also been in the stage of exploration and development, and many achievements have been made. Through the investigation, it is found that the number of literature review on algorithmic composition is relatively large, but the literature review on music generation based on deep learning, especially based on generating confrontation network, is relatively missing.

## 2. Related Work

As early as the 1990s, the literature first proposed the idea based on confrontation generation. The author trained the predictor to judge the input data mode, and let each data minimize its predictability, forming the simplest confrontation competition learning mode. In 2014, P. Shamsolmoali [18] et al. formally put forward the concept of generating countermeasure network, using the confrontation competition of generator model and discriminator model to realize semi supervised learning, which opened up a new field for the research of data generation. The biggest difference between GANs and the traditional generation model is that in the process of data training, it is both unified and antagonistic. The optimization directions of generators and discriminators are different from each other, forming a competitive relationship, but their optimization calculation depends on each other's output to form a unified system [19]. In the confrontation training mode, the generator no longer directly learns the distribution from the training data set, but indirectly iteratively learns through the optimization direction given by the discriminator to generate fake samples that confuse the false with the true. Compared with the traditional unsupervised learning models such as self coding and autoregression, GANs has the advantages of fast calculation speed, better sample quality, strong expansion flexibility and so on [20]. Generally speaking, the production process of intelligent music needs to minimize the workload of human intervention. This production method can generate music automatically or semi automatically. The output results should not only meet the basic prior knowledge of music theory, but also have some algorithm creativity. The literature discusses that this process is to independently produce continuous audio signals or discrete symbol sequences from the computational model, and these signals and sequences must meet the music theory architecture.

Subsequently, a large number of theoretical and technical research results of generative countermeasure networks came out one after another, and some of them played a milestone role in promoting the overall research progress of generative countermeasure networks. The verification model of the original GANs is realized by multi-layer perceptron MLP, and the generation quality is poor [1]. The literature proposes DCGANs, and the generator and discriminator are realized by deep convolution network respectively, so as to ensure the engineering implementation of GANs in the field of graphics generation. In order to improve the training stability of GANs and make the output data have a certain controllable directivity, the literature proposes the conditional generation confrontation model CGANs [21]. On this basis, the literature adds a supervised learning classification task to GANs to form ACGANs to improve the generation quality of the model. In order to further explore the training stability of GANs, some targeted training skills are added in the training process of GANs to form improved GANs. The literature improves the loss function of GANs from the mathematical principle, so that GANs can better narrow the distribution of generated data and training set data [22]. This research work has played an important role in the development of GANs technology, and also made a series of application achievements in many task fields, especially in the direction of graphics and images. At present, there are many research works on GANs with wide coverage. This paper investigates and classifies the existing achievements from the key technical level and application level. It should be noted that the same work may contain improvements in multiple directions [23].

There are some problems caused by the loss function in the training process of the original GANs. For example, it is proved by theory that the original loss function will cause the gradient to disappear when the coincidence between the generated data distribution and the training set data distribution is not enough [24]. This problem cannot be improved by simple model structure optimization. Therefore, some work has improved the loss function, so as to improve the generation quality of GANs. Firstly, it is proved that the distance measurement between distributions based on KL distance and JS distance is discontinuous, which leads to the instability of discriminator training. The sample data generated by GANs is constrained by confrontation training and tries to fit the distribution of real data sets [25]. However, there is no guidance of conditional control in the whole training process, and the randomness of generated data is great, which also indirectly leads to the instability of training. The literature integrates conditional control into countermeasure training, uses conditional vectors to control the attributes of generated samples, and indirectly introduces the decoupling input of conditional variables. On the basis of CGANs, the literature uses discriminators for supervised learning, classifies samples, further enhances the conditional constraints, and further improves the quality of generated samples [26]. The proposal of generative countermeasure network not only presents a new network model architecture, but also provides a framework of generative countermeasure training idea. After a certain expansion of the framework, it can be combined with other existing model methods to generate samples for specific fields.

## 3. Generative Confrontation Mechanism and Its Important Derivative Model in the field of Music Generation

### 3.1. Generation Confrontation Mechanism

The generative countermeasure mechanism consists of two sub networks: generator and discriminator. The task of generator *F* is to make the model fit the real training data distribution as much as possible, and discriminator *B* is committed to distinguishing whether the input data comes from real data or false data manufactured by the generator. In the training process, they continuously improve their ability, and finally achieve an ideal state of Nash equilibrium, so that the data generated by the generator is as close to the real data distribution as possible. The basic loss function of GAN is:

(1) $$\begin{array}{c} \underset{B}{\min}\;\underset{F}{\max}\;V\left(B,F\right)=\frac{T_{Z-Pz\left(z\right)}\left[\ln\left(1-B\left(F\left(z\right)\right)\right)\right]}{E_{x-\left(z\right)}\int \left[\prod D\left(x\right)\right]\text{d}x}. \end{array}$$

**Figure 1:**
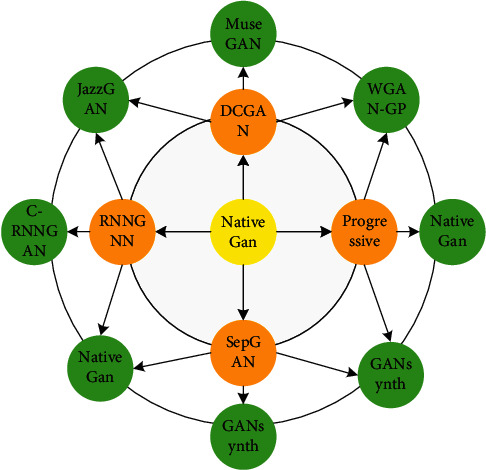
Generation model of GAN and its derived music.

In theory, the generator and discriminator can be realized by any differentiable function, which is not limited to multi-layer perceptron or convolutional neural network. GAN and its derived music generation model are shown in Figure 1.

## 4. Generation of Derivative Models of Countermeasure Networks

### 4.1. WGAN

Zhang [21] et al. have studied and discussed various problems existing in the original generated countermeasure network. There are two main reasons for these problems in the original generated countermeasure network: 1. Unreasonable distance measurement of equivalent optimization (KL divergence and JS divergence); 2. There is no intersection or overlap between the data distribution generated after the random initialization of the generator and the actual data distribution, which can be ignored. However, it is not suitable to use JS divergence to measure the distance between the disjoint parts. Theoretically, this is also the main reason for the instability of the original generation anti network training. Therefore, Wasserstein GANs was put forward by homeopathy (WGAN). WGAN has made a good improvement on the problem of unstable training of generation countermeasure network model. WGAN uses Wasserstein distance as an alternative to JS divergence to measure the distance between generated data distribution and real data distribution, and solves the two problems of unstable training and unable to provide numerical indicators to measure the training process. Compared with KL divergence and JS divergence, Wasserstein distance can still be used to measure the distance between the generated data distribution and the real data distribution when there is no intersection or the intersection part can be ignored.

Before introducing the basic principle of WGAN, we first need to understand the concept of Lipschitz continuity, that is, for a continuous function *f* (*x*), if there is a constant *k* > 0, any two elements *x* and *x*0 in the definition domain meet:

(2) $$\begin{array}{c} \int_{x_{0}}^{x}g\left(x\right)+g\left(x_{0}\right)\leq F\sqrt{{\left(x-x_{0}\right)}^{2}-4x}+\sqrt{x^{2}-4x}. \end{array}$$

Wasserstein distance can not only reflect the distance of two nonoverlapping or negligible overlapping distributions, but also provide a meaningful gradient, that is, it is smooth. However, it is impossible to solve the lower bound directly. Therefore, the Wasserstein distance can be changed into the following form according to Kantorovich Rubinstein duality:

(3) $$\begin{array}{c} \underset{B}{\min}\;\underset{F}{\max}\frac{\prod \left[E\int \left(x\right)\right]+\sum_{x-p}E\left(\tilde{X}\right)}{\left|x-\tilde{X}\right|}. \end{array}$$

### 4.2. WGAN-GP

**Figure 2:**
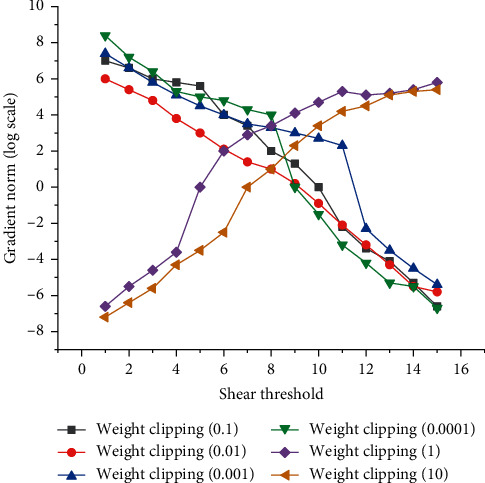
Shear threshold gradient experimental diagram.

In view of the problems of unstable training and slow convergence in the generated countermeasure network, Wasserstein GANs proposed by Yang [22] et al. has made a good improvement, but sometimes the generated samples are still bad or the convergence fails. Then, scholars such as Shi [23] proposed that the main reason for the failure of bad sample generation or convergence based on WGAN is that the weight clipping method is used to meet the Lipschitz continuity condition in the implementation of WGAN, which will lead to bad training behavior. In this regard, scholars such as Ishaan Gulrajan proposed WGAN-GP (improved training of Wasserstein GANs), that is, the improvement of WGAN network model based on gradient penalty, which uses gradient penalty instead of weight clipping to meet Lipschitz continuity condition. In this case, the extreme distribution of parameter weights cannot give full play to the generalization ability of deep neural network. In addition, weight shearing can easily lead to gradient explosion or gradient dispersion. The reason is that when weight shearing is carried out, if the shear threshold is set too small, the gradient will gradually decrease through each layer of network, and then decay exponentially until the gradient disappears; When the shear threshold is set too large, the gradient will gradually increase after each layer, and then the gradient will explode, as shown in Figure 2.

Therefore, only when the threshold is set at a balanced position can the normal gradient return be guaranteed. However, due to the narrow balance area, the parameter adjustment work becomes very troublesome.

### 4.3. CT-GAN

Aiming at the problems of difficult training and slow convergence of WGAN, WGAN-GP improves WGAN and uses the gradient penalty method instead of the weight clipping method to meet the Lipschitz continuity condition. WGAN-GP makes network training faster and convergence faster, but WGAN-GP also has defects. According to what is mentioned in the paper, the difference between gradient penalty and weight clipping is that gradient penalty can never be punished to every place through gradient penalty term in a limited number of training times, and the result is that gradient penalty term GP only works on sampling point *x*. The important parts of the support set cannot be checked at all, especially the observed data points and the basic manifold supporting the real data distribution pr.

In addition, CT-GAN does not only focus on one specific data point at a time, but sets regularization on a pair of data points drawn near the manifold according to the most basic definition of Lipschitz continuity. In particular, CT-GAN perturbs each actual data point *x* twice, and uses the Lipschitz constant to limit the difference between the response of the discriminator to the set data point.

**Figure 3:**
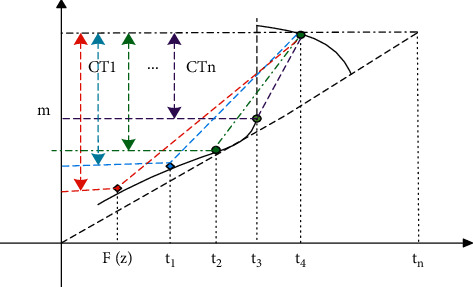
Main idea diagram of CT-GAN.

Figure 3 describes the main idea of CT-GAN. Because the gradient penalty often fails to verify the continuity of the region near the real number *x*, the discriminant function can freely violate the Lipschitz continuity. Therefore, on the basis of gradient penalty, CT-GAN uses the inspection of two disturbed Lipschitz continuity conditions near any observed actual data point, so as to alleviate the problem of WGAN-GP.

### 4.4. Generative Model and Discriminant Model

**Figure 4:**
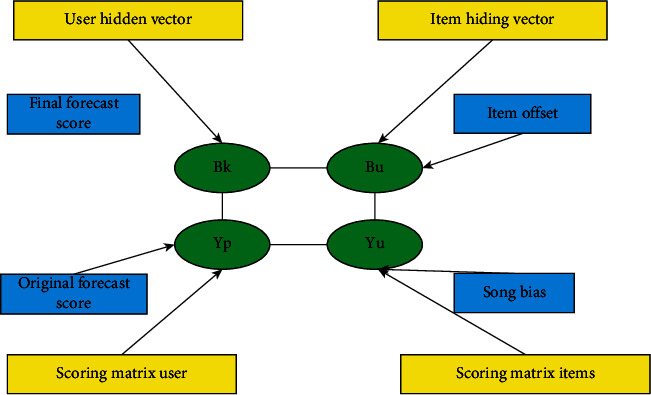
Generator simulation.

The main structure of the generator model (discriminator model) is shown in Figure 4. It can be seen that its structure is relatively simple. The main reason is the repeated iterations in the training process. Each round of training is equivalent to training dozens of generators and discriminators, and each iterative operation needs to store the neural network parameters of the intermediate process, so only one layer of neural network is used here. Moreover, there is a problem of parameter feedback in the training process of generator and discriminator. The trained discriminator parameters are superimposed into the generator parameters as negative feedback. Too complex network will lead to poor feedback process.

## 5. Generation Experiment and Analysis of Intelligent Music Generating Confrontation Mechanism

### 5.1. Data Set and Pre-Processing

In the experiment of music generation in this paper, the MIDI file of lakh MIDI data set (LMD) is selected to generate music containing the following five tracks: bass, drum, guitar, piano and strings.

LMD is used because it provides a rich and real collection of MIDI files and some related metadata. The lakh MIDI dataset is a collection of 176581 unique MIDI files, of which 45129 have been matched and aligned with the entries in the Millennium Song Dataset. The goal of Lakh MIDI dataset is to facilitate large-scale music information retrieval, including symbols (only MIDI files) and audio content-based (using information extracted from MIDI files as annotations for matching audio files). Lakh MIDI data sets have many different versions, some of which are subsets and some of which are obtained after processing. LMD-full is the complete set of Lakh MIDI data sets. Each file is named according to its MD5 checksum. However, since invalid MIDI files are not deleted from this collection, it contains thousands of files that may have been damaged; LMD matched is a subset of LMD-full containing 45129 files, which matches the entries in the Millennium Song Dataset.

However, in this experiment, the music in piano roll format is generated instead of MIDI format, so Lakh MIDI data set will not be directly used as the training data set of this multi track music generation experiment, but a series of pre-processing steps will be performed on Lakh MIDI data set before training, Thus, the LMD piano roll data set needed by the experiment is obtained, and then it is put into the network for training to generate multi track music. In the next two sections, we will first introduce the data representation of this experiment-Piano Roll format, and then introduce the pre-processing process of data set.

### 5.2. Data Pre-Processing

According to experience, there are some defects in using LMD directly for music generation, mainly because of the mapping between track and MIDI channel. Because these MIDI files are usually user generated, the mapping is error prone. Therefore, before the experiment, the following three data cleaning steps are performed to pre-process the data.

Divide the different tracks of each MIDI music file in LMD : This paper divides each MIDI file into five tracks: Bass, drum, guitar, Piano and string. The music generated by the experiment also includes these five tracks. This is because there is no clear method to identify and distinguish the track where the main melody and chord are played. Therefore, the music track cannot be divided into melody, rhythm and drum.

Select “rock” songs and merge tracks with sparse notes. Firstly, this paper mainly selects the music marked as “rock” songs, because this category generally contains the above five tracks, which can reduce the number of blank sections in these tracks. Although songs with genre tags are filtered, some tracks still tend to play only a few notes in the whole song. This increases data sparsity and hinders the learning process. This paper combines the tracks with sparse notes into the most similar one of the above tracks. Because string instruments are the most diverse among a few instruments, instruments that are difficult to classify are merged into the track where string instruments are located.

### 5.3. Experimental Results and Analysis

After training, a large number of music clips are generated using the MCT-GAN model in this paper. After listening, it is found that most of the generated music is smooth and has a certain artistic aesthetic effect. Here, randomly select the generated music clips as the result display. Firstly, the generated multi track music is presented in the format of score. The generated music file is converted from npz format to mid file format by using Python library pypianroll, and then the mid file is expanded in the format of music score by using Cubase software. In order to make the music score clearly displayed, only four sections of music fragments are presented, and separate music scores are derived for each section. Therefore, the music score includes four sections of generated music.

**Figure 5:**
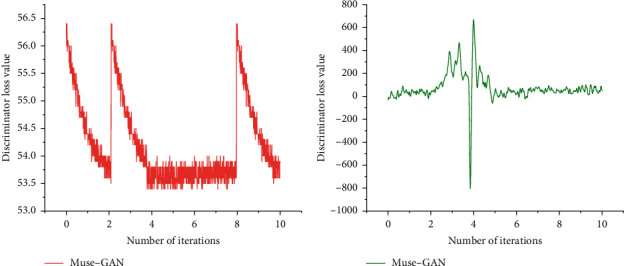
Comparison diagram of convergence curve of discriminator loss.

As mentioned above, MCT-GAN has made two innovations in the previous music generation model. 1. Use CT-GAN as the generation confrontation model. 2. Propose a new time structure model. Muse GAN is a multitrack music generation network based on WGAN-GP. Therefore, this paper compares the MCT-GAN experiment with Muse-GAN experiment, so as to analyze the experimental effect of MCT-GAN proposed in this paper. According to the experimental results, the generation results obtained by MCT-GAN and Muse-GAN experiments are compared as follows:

Firstly, the training loss of the discriminator at different training iteration points is obtained experimentally. Therefore, the convergence curve of discriminator loss can be obtained and compared with the convergence curve of Muse GAN. The results are shown in Figure 5.

From the two convergence curves of Muse GAN and MCT-GAN in Figure 5, it can be found that compared with Muse-GAN based on WGAN-GP, MCT-GAN with increased CT penalty term converges faster and the training process is more stable.

**Figure 6:**
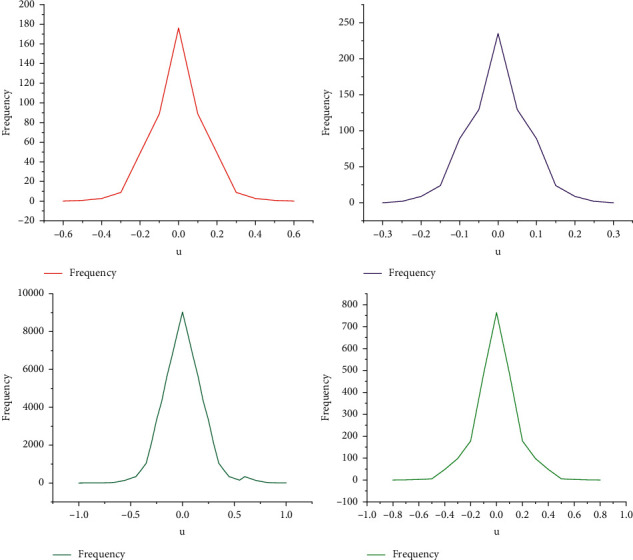
Parameter distribution histogram.

Through the experiment, the distribution histograms of discriminator parameters in the two experimental iterations can be obtained respectively, as shown in Figure 6.

From the comparison in the figure, it can be found that the interval range of the parameter distribution obtained from the music generation experiment based on MCT-GAN is significantly smaller than that obtained from the Muse-GAN experiment, which shows that compared with the Muse-GAN based on WGAN-GP. The MCT-GAN with consistency penalty proposed in this paper is less prone to over fitting. In conclusion, through experimental comparison, MCT-GAN is better than Muse-GAN in stability, convergence speed and over fitting. These indicators can be calculated from the training (actual) data and the generated data to obtain the corresponding indicator values, and by comparing the values calculated from the actual data and the generated data, the effect of the generator can be understood. Therefore, the idea of the index evaluation method is consistent with the idea that the distribution of real and fake data in GAN should be close.

Muse-GAN designed an index evaluation method to evaluate the generated results. Therefore, this paper uses this method to compare Muse-GAN and MCT-GAN. The following indicators are defined in Muse-GAN, where EB, UPC and QN are in track evaluation indicators, and TD is inter track evaluation indicators. These indicators can be calculated from the training (actual) data and generated data, and the effect of the generator can be understood by comparing the values calculated from the actual data and generated data. Therefore, the idea of index evaluation method is consistent with the idea that the distribution of real and false data in GAN should be close.

**Figure 7:**
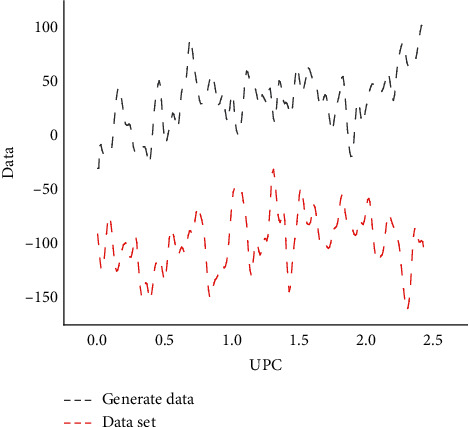
UPC index curve of stringed instruments.

Figure 7 shows the change of UPC index value of the track where the string instrument is located during the experiment. The blue line in the figure is the UPC index value of the string instrument in the training set. It can be found from the figure that *G* can learn this index during training, which also shows that these index values can be used to preliminarily evaluate the generation results of the model before subjective evaluation.

**Figure 8:**
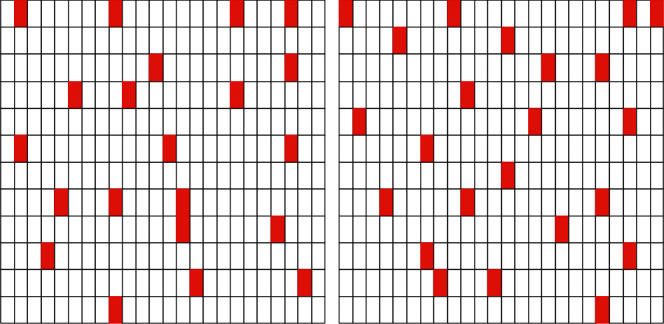
Comparison of blank sections.

In addition, this paper also randomly selects several sections of piano volumes as a comparative display, as shown in Figure 8. Figure 8a is a piano volume diagram for generating music using T3 as the time structure, and Figure 8b is a piano volume diagram for generating music using T1 as the time structure. The sections framed by the red box (or thick box) in the figure represent empty sections. From the comparison of the two figures, it can be seen that there are fewer blank sections using T3 as the time structure than using T1, which shows that the generation effect of MCT-GAN model using T3 as the time structure is improved compared with Muse-GAN.

In conclusion, using the index evaluation method to compare the experimental results, it is found that compared with the music generation experiment based on Muse-GAN. The MCT-GAN proposed in this paper has improved the effect of generating blank bars, generating note quality and generating music harmony. In addition to comparing the results, this paper also uses a subjective manual evaluation method. Select 80 test listeners and listen to 8 music clips for each tester. The specific proportion of the two is random. Then, after listening to each piece of music, the tester asked them to identify the source of the music and generate the music from the given three options (A. music; *b*. Real music; *c*. Finally, according to the selection results of all testers, the accuracy rate (i.e. the probability that the selection item is correct) and the confusion rate (i.e. the probability that *c* is selected) of each audience are obtained.

The results showed that 72.5% of the testers could not distinguish the generated music from the real data, that is, the number of people with a correct rate of less than 50%. Moreover, among the testers whose accuracy is in the range of 0%–25%, most of the testers cannot distinguish after listening to the music, and finally choose the uncertainty C; After the test, when interviewing the testers whose accuracy is in the range of 75%–100%, it is found that some testers have guessing behavior in discrimination. Therefore, from the results of manual evaluation, it can be found that the music production method proposed in this paper is effective.

## 6. Conclusion

With the increasing improvement of material living standards, people's cultural and entertainment life is becoming richer and richer, and people's demand for spirit is increasing day by day. However, in the field of artificial intelligence, generating real and aesthetic works of art has always been considered a challenge. With the rapid development of artificial intelligence technology and the development of deep learning technology in recent years, great progress has been made in image, audio and text generation. In particular, the generation experiment is carried out with the very popular generation countermeasure network, and remarkable results are achieved. Therefore, this paper studies the existing theoretical results. Finally, based on the improved derivative model CT-GAN of generation countermeasure network, the generation experiment of intelligent music is completed.

Because the experimental research design of this paper is based on the popular deep learning model to generate countermeasure network, this paper first briefly introduces the development background and basic concepts of deep learning. Then, the basic principle of generating countermeasure network is understood in detail, and the advantages and disadvantages of generating countermeasure network are analyzed. Then, understand an improved model Wasserstein GANs proposed for the problems of generating countermeasure network, and learn its basic principle. Although WGAN has improved Gan network, there are still some problems. Therefore, this paper also studies WGAN-GP, which is an improved model of WGAN network based on gradient penalty. For better training effect, this paper also makes a detailed study on the latest improved model CT-GAN based on consistency penalty term, which is proposed to solve the problems of WGAN-GP.

Learning and improving the existing music generation model based on GaN. When studying the multitrack music generation based on generation countermeasure network, this paper studies and analyzes three models. Based on them, the multitrack music generation method based on CT-GAN is proposed in this paper, which mainly improves the existing music generation model based on GAN. The music generated in this paper only contains five tracks: bass, drum, guitar, piano and string. However, some music creations in real life contain more different tracks. Therefore, in the follow-up work, we can consider using richer music data sets to generate music containing more tracks, so as to make the generated music more diverse. This paper realizes the multitrack music generation experiment based on CT-GAN, which can generate 4 bar music including 5 tracks. In the follow-up work, this music generation can be extended to music composition related applications to assist composers or music lovers in more convenient and diverse music creation. During data pre-processing, in order to reduce the number of empty sections contained in music data and deal with the data imbalance problem that increases data sparsity and hinders the learning process due to several notes played in some tracks in the whole song, the corresponding piano volumes are added and similar musical instruments are combined into one of the above tracks. However, this will bring noise to the data and affect the generation effect.

## Data Availability

The data used to support the findings of this study are available from the corresponding author upon request.
